# Theoretical study of the photoisomerization of 1,2-bispyrazinyl-ethylene and the halogen ion salts of 1-Pyrazinyl-2-(4’-methylpyrazinyl)ethylene

**DOI:** 10.1007/s00894-024-05881-9

**Published:** 2024-03-20

**Authors:** Adebayo A. Adeniyi, Jeanet Conradie, Karel G. Von Eschwege

**Affiliations:** 1 Department of Chemistry, University of the Free State, PO Box 339, Bloemfontein, 9300 South Africa; 2 Department of Industrial Chemistry, Federal University Oye-Ekiti, Oye, Ekiti Nigeria

**Keywords:** Excited state Potential Energy Surface, Intersystem crossing, Internal conversion, Phosphorescence, Fluorescence, Charge transfer

## Abstract

**Context:**

It has been reported that photoexcitation of azastilbene compounds like E-1,2-bispyrazinyl-ethylene (bpe) can undergo E → Z photoisomerization of its quaternary salts via the excited triplet state. However, experimentally it is possible to get low fluorescence and photoisomerisation quantum yields in a state with higher internal conversion than intersystem crossing. We modelled bpe and its methylated derivative (bpeMe), as well as its quaternary halogen salts (bpeMeX with X = F^−^, Cl^−^, Br^−^ and I^−^) to study levels of fluorescence, phosphorescence and excited state potential energy surfaces (PES). Results support experimental observations of molecules where the anion of a salt is an efficient electron donor, that molecules with weak electron-donating anions like Cl^−^ to give increased fluorescence and photoisomerization, as compared to molecules with stronger electron-donating anions like I^−^, which are dominated by competing electron transfer. The fluorescence of bpeMeF and bpeMeCl was found to be stronger than bpeMeBr and bpeMeI. A deep well in the triplet excited state of bpeMeI is considered responsible for the decreased photoisomerization, compared to what was experimentally observed for bpeMeCl. Uniquely, the bpeMeI molecule is characterised by near-zero splitting of the s1 and t1 excited states that can enhance charge transfer. The quaternary salt of bpeMe with stronger electron-donating Br^−^ anion was observed to undergo fluorescence and phosphorescence at much lower energy compared to those with weak electron-donating F^−^ and Cl^−^ anions. This research shows how to control the excited state fluorescence, phosphorescence and isomerization of quaternary halogen salts of methyl derivatives of 1,2-bispyrazinyl-ethylene, which aids experimental design where excited state isomerization is considered.

**Methods:**

Geometry optimization, molecular electrostatic potential (MESP), and time dependent density functional theory (TDDFT) calculations were conducted utilizing Gaussian 16 with the B3LYP functional and the 6–31 + G(d,p) basis set. The minimum energy path (MEP) for the E to Z isomerization of the molecules was established employing the Nudged-Elastic-Band (NEB) method, implemented in Orca 4.2. Precise energies of the E → Z isomerization reaction path were determined employing CASSCF and a more accurate multireference method, NEVPT2.

**Supplementary Information:**

The online version contains supplementary material available at 10.1007/s00894-024-05881-9.

## Introduction

**Scheme 1:**
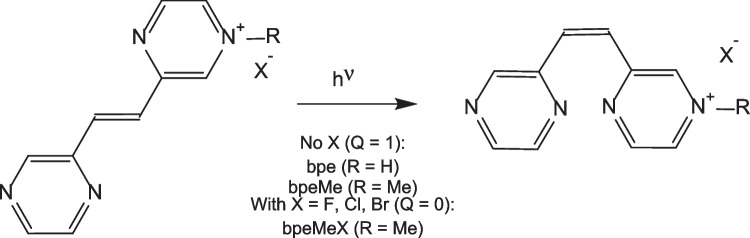
Structures and abbreviations used, of E and Z isomers of molecules of the present study. Q = charge

Photo-excitation of azastilbene compounds undergo photoisomerization, exhibiting low fluorescence and photoisomerization quantum yields in states with higher internal conversion than intersystem crossing [1]. Internal conversion (IC) may thus be one of the major deactivation processes in the excited singlet state. The photochemical and photophysical behaviour of azastilbenes are affected by the n,π* states introduced by heteroatoms [1]. Efficient internal conversions (IC) or intersystem crossings (ISC) are found in many azastilbenes because of the vibronic perturbation of the lowest π,π* or n,π* singlet state by close-lying n,π* or π,π* states [2]. E → Z photoisomerization of quaternary salts of E-1,2-bispyrazinyl-ethylene (bpe) are reported to proceed through the excited triplet state [1], see structure in Scheme 1. The halogen anion serve as a weak source of electron donor [1]. Photoisomerization of E → Z was found to be efficient in the 1-pyrazinyl-2-(4’-methylpyrazinyl)ethylene (bpeMe) salt when containing a weak electron-donating anion like Cl^−^ (bpeMeCl) (compared to stronger electron-donating anions like Br^-^ and I^-^). Here, inter-system crossing (ISC) to the excited triplet state from the excited singlet state plays an important role compared to those with I^−^ (bpeMeI) where electron transfer from the singlet state competes with ISC. In the presence of a strong electron-donating anion like I^−^, deactivation of the excited singlet state is dominated by electron transfer that competes efficiently with both the fluorescence and photoisomerization yields of the neutral E-bpeMeI salt [1].

The study of photochemistry of quaternary salts of azastilbene derivatives in the presence of halogen anions is important since it has several applications in environmental, physical and biological systems. These studies were used to show how the presence of halogen anions affect the phototoxicity of anthracene-based chromophores, as well as its effect on inhibition of DNA damage cleavage (I^−^ > Br^−^ > F^−^) [3]. Also, halogen anions were shown to be suitable electron-donors in the formation of electron-donor–acceptor (EDA)-complexes during excited state transformation of quaternary derivatives of pyridinium, which are known to have several valuable applications in both biological and physical systems [4].

**Fig. 1:**
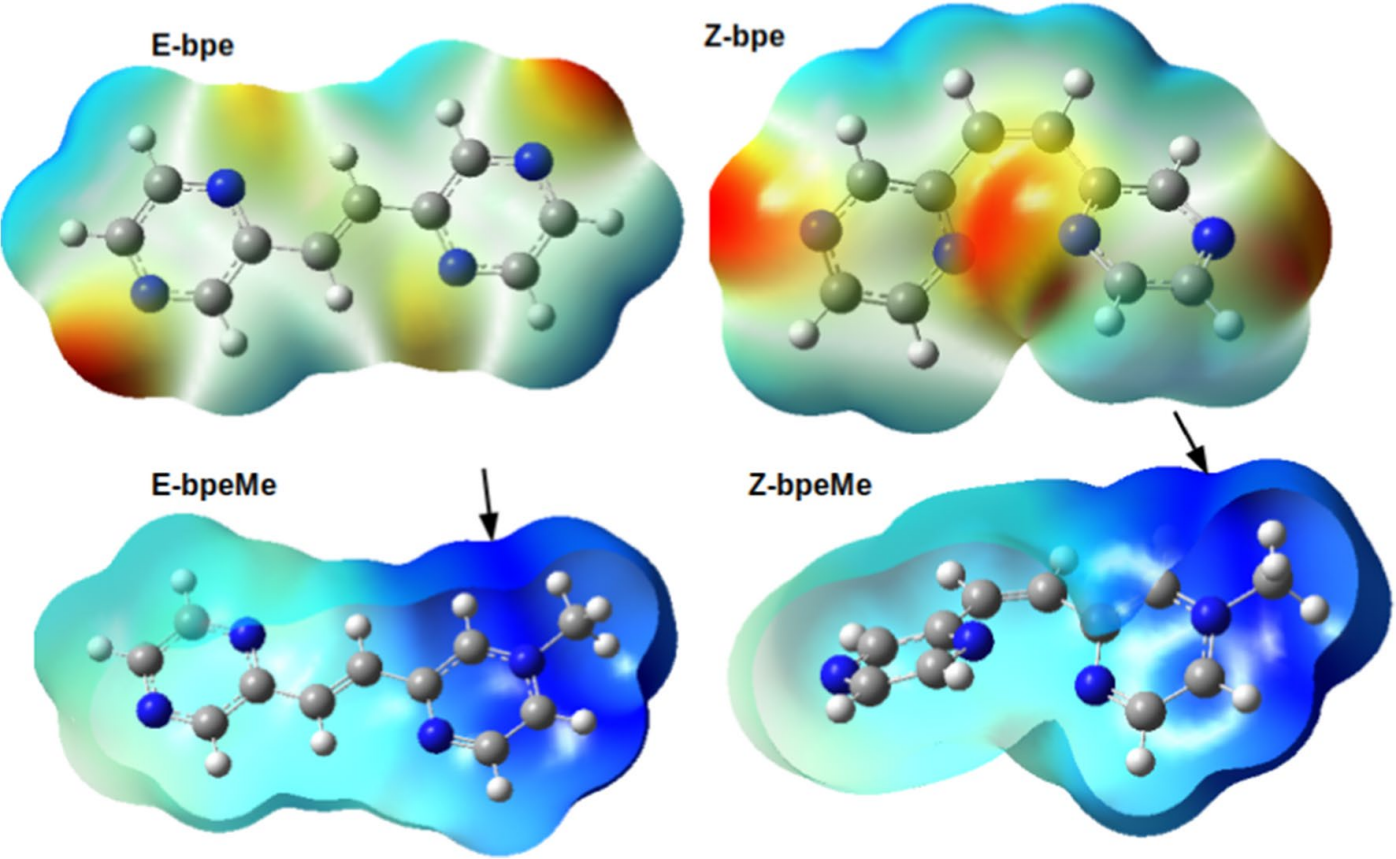
Molecular electrostatic potential (MESP) surfaces of the optimized geometries of the E and Z isomers of bpe and bpeMe. Most electrophilic areas for anion interaction of bpeMe are indicated by arrows. Atom colour codes: N (blue), C (grey), H (white)

The structures of the cations, bispyrazinyl-ethylene (bpe) and its derivative bpeMe ion are shown in Fig. 1. In the halogen salts of bpeMe, the halogen anion serve as a weak source of electron donor [1]. Literature reports show that molecules with strong electron-donating anions like I^−^ and Br^−^ are associated with higher electron transfer leading to lower fluorescence quantum yields Φ_f_ and photoisomerization quantum yields Φ_E→Z_. This is contrary to bpeMe cations with weak electron-donating anions like F^−^ and Cl^−^. We studied the photophysical and E → Z photochemical properties of the cations bpe and bpeMe to elucidate the effect of the positive charge and the presence of strong (Br^−^ and I^−^) and weak electron-donating halogen anions (F^−^, Cl^−^) that form the corresponding quaternary salts (bpeMeX with X = F^−^, Cl^−^, Br^−^ and I^−^) of bpeMe. In this study, differences in electron transfer, excitation energy, photophysical properties (fluorescence and phosphorescence) and nature of the excited state reaction path for the E → Z photoisomerisation of the bpe cation, its derivative the bpeMe cation and their halogen salts were computed (Scheme 1). The CASSCF method was applied, being a useful method for computing excited state properties of molecules. CASSCF was applied in several studies, eg. in dual fluorescence of 2-(20-hydroxyphenyl) [5], and excited-state decay of 8‑Methoxy-4- methyl‑2H‑benzo[g]chromen-2-one [6].

## Computational method

The geometries of the E and Z isomers of the bpe and bpeMe (charge Q = 1) molecules were modelled and optimized to its local minima. The molecular electrostatic potential (MESP) surface was computed for the optimized structure of bpeMe to know the best electrophilic centre for the introduction of the halogen anions that act as nucleophiles. Halogen anions were thus introduced to interact with the bpeMe cation, and named bpeMeF, bpeMeCl, bpeMeBr and bpeMeI (all Q = 0). Initial optimization and MESP was carried out using Gaussian 16 [7] with the B3LYP functional and the 6–31 + G(d,p) basis set.

To study the reaction path for the isomerisation of the molecules from their E to Z isomers, the Nudged-Elastic-Band (NEB) method [8, 9] as implemented in Orca 4.2 [10, 11] was applied to locate the minimum energy path (MEP). In all the studied molecules, eight geometries were extracted along the isomerisation MEP, which include the initial structures of the E and Z isomers.

Changes in energy along the sample reaction path for the ground and excited states were computed using CASSCF and then corrected with internally implemented multireference perturbation theory, i.e. N-Electron Valence State Perturbation Theory (NEVPT2) [12, 13]. The advanced CASSCF and perturbation methods that were used had def2-TZVP [14] as basis set, as implemented in Orca.

Computation of other molecular excited state properties was done by using the TD-DFT method with B3LYP functional method and 6–31 + G(d,p) basis set. MESP analysis was also done with the same functional and basis set, while rendering of MESP isosurfaces was done with VMD [16] and Multiwfn [17, 18]. The CASSCF, NEVPT2, TD-DFT and MESP computations were carried out in acetonitrile as solvent (the experimental solvent used for photoisomerization of bpeMeX) with the SMD solvation model [15].

## Results and discussion

### E to Z isomers reaction path

The molecular electrostatic potential (MESP) of the optimized E and Z isomers of bpe and bpeMe molecules were computed with the surfaces shown in Fig. 1. MESP is associated with electronic density and serves as useful descriptor for identifying areas for hydrogen-bonding interactions, nucleophilic reactions and electrophilic attack [19, 20]. The MESP surfaces of the optimized geometries of the E and Z isomers of bpe and bpeMe are shown in Fig. 1. Methyl group presence gives rise to a positively charged bpeMe molecule with a strong electrophilic field around the nitrogen atom that carries the methyl group, which then is available for anion interaction. The anions were introduced to the bpeMe molecules through the most electrophilic centres around the nitrogen atom that bears the methyl group.

**Fig. 2:**
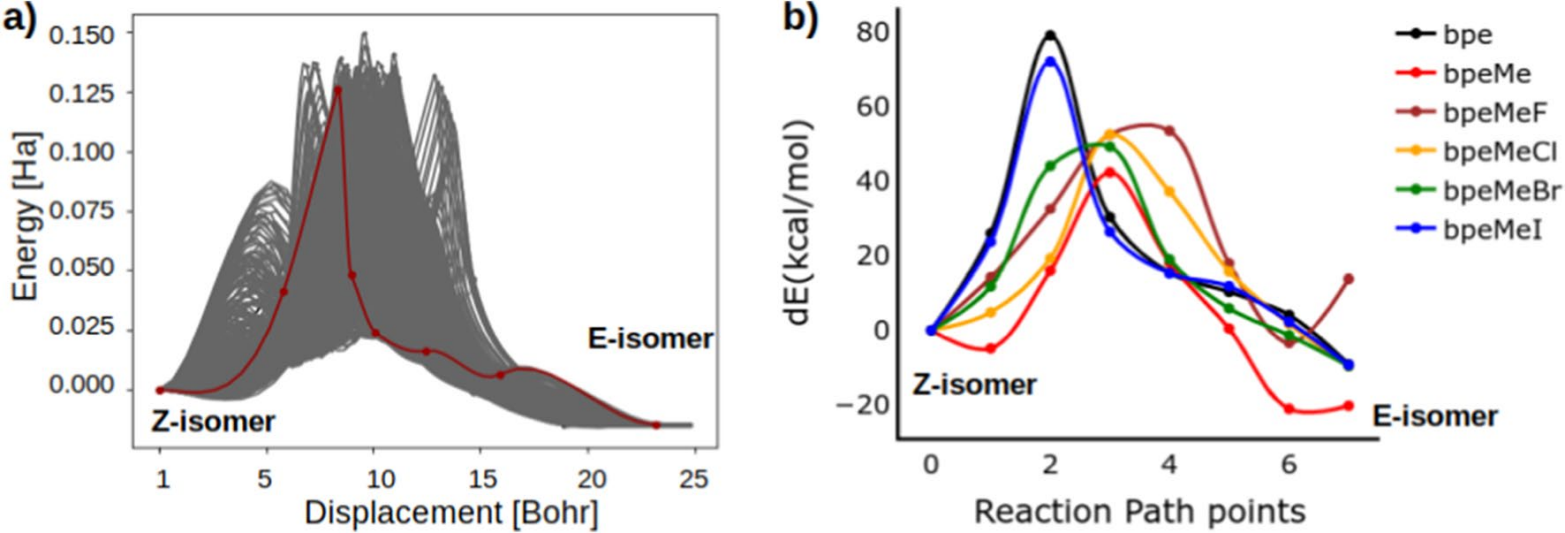
(**a**) The minimum energy reaction path (in red) for E to Z isomerisation of bpe, using the NEB method. The eight points selected for further study are indicated as dots on the reaction path, red. (**b**) Reaction path plots were obtained from NEB analyses for all molecules. The energy of the Z-isomer is taken as 0. dE = change in energy

The reaction path for the E to Z isomerisation obtained from the NEB method for bpe is shown in Fig. 2. After sampling several possible conformational changes along the E to Z isomerisation path, the best path was chosen based on the convergence of NEB to the minimum energy path as indicated in Fig. 2. In each of the molecules eight points indicated by dots along the minimum energy path, were selected for further computational studies. Reaction paths along the constructed MEP for all the other molecules are shown in Fig. 2. The predicted energy barrier from NEB analysis shows that the energy barrier for the bpe cation and the bpeMeI neutral molecule, is higher than the rest of the molecules in the order:

$$\mathrm{bpe}\:>\:\mathrm{bpeMeI}\:>\:\mathrm{bpeMeF}\:>\:\mathrm{bpeMeCl}\:>\:\mathrm{bpeMeBr}\:>\:\mathrm{bpeMe}.$$

The structure of the E-isomer has a lower energy compared to the Z-isomer in all the molecules except for the fluor salt, bpeMeF, see Fig. 2(b).

### Excited state structural change

**Fig. 3:**
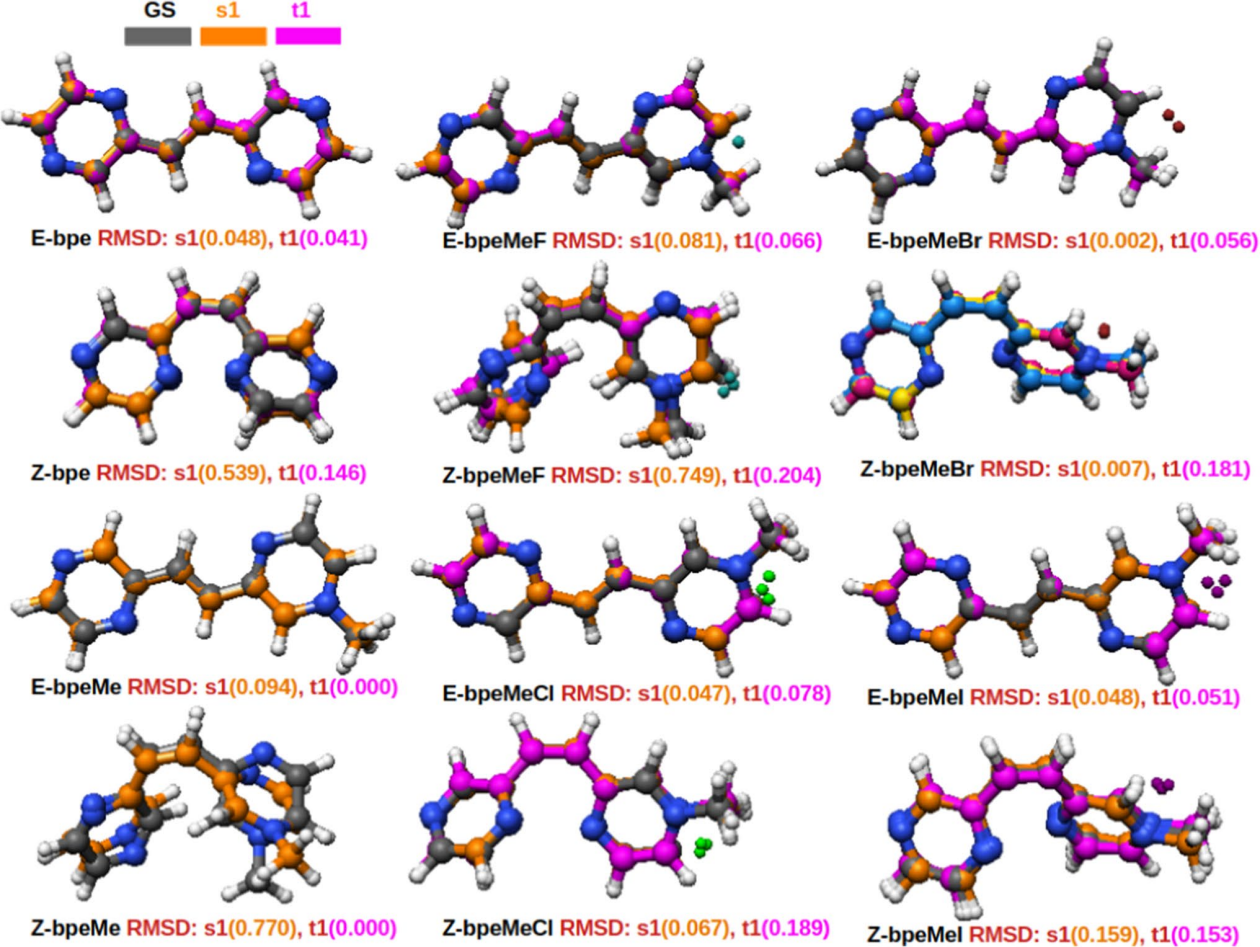
Ground state (GS), first singlet excited state (s1) and first triplet excited state (t1) structures of the B3LYP/6–31 + G(d,p) optimized geometries. RMSDs of the backbone atoms (carbon and nitrogen atoms) of each of the excited state structures from the GSs are indicated

To explore excited state structural changes, DFT (B3LYP/6–31 + G(d,p) was used to optimize the geometries of the E and Z isomers in their ground states (GS), first excited singlet (s1) and triplet (t1) states. Geometry changes of excited state structures derived from ground state structures are shown in Fig. 3, as the RMSD from GS structures. The RMSD values of s1 and t1 of the GS structures clearly show structural changes in both the s1 and t1 excited states of the molecules, as compared to their ground states. The RMSDs of the E-isomers are relatively low compared to that of the Z-isomers. The RMSDs clearly show more significant changes in geometries in the s1 state compared to the t1 state in the bpe and bpeMe molecules and the fluorinated molecules. The opposite is observed in the Cl^−^, Br^−^ and I^−^ anions, with larger geometry changes in the t1 state than in the s1 states. The most significant change in geometry is observed in the s1 excited state of the Z-bpeMe (with root-mean-square deviation RMSD = 0.770 Å) and Z-bpeMeF (RMSD = 0.749 Å) molecules, as seen in the rotation of one of the two rings.

### Ground and excited state E → Z isomerisation reaction paths

**Fig. 4:**
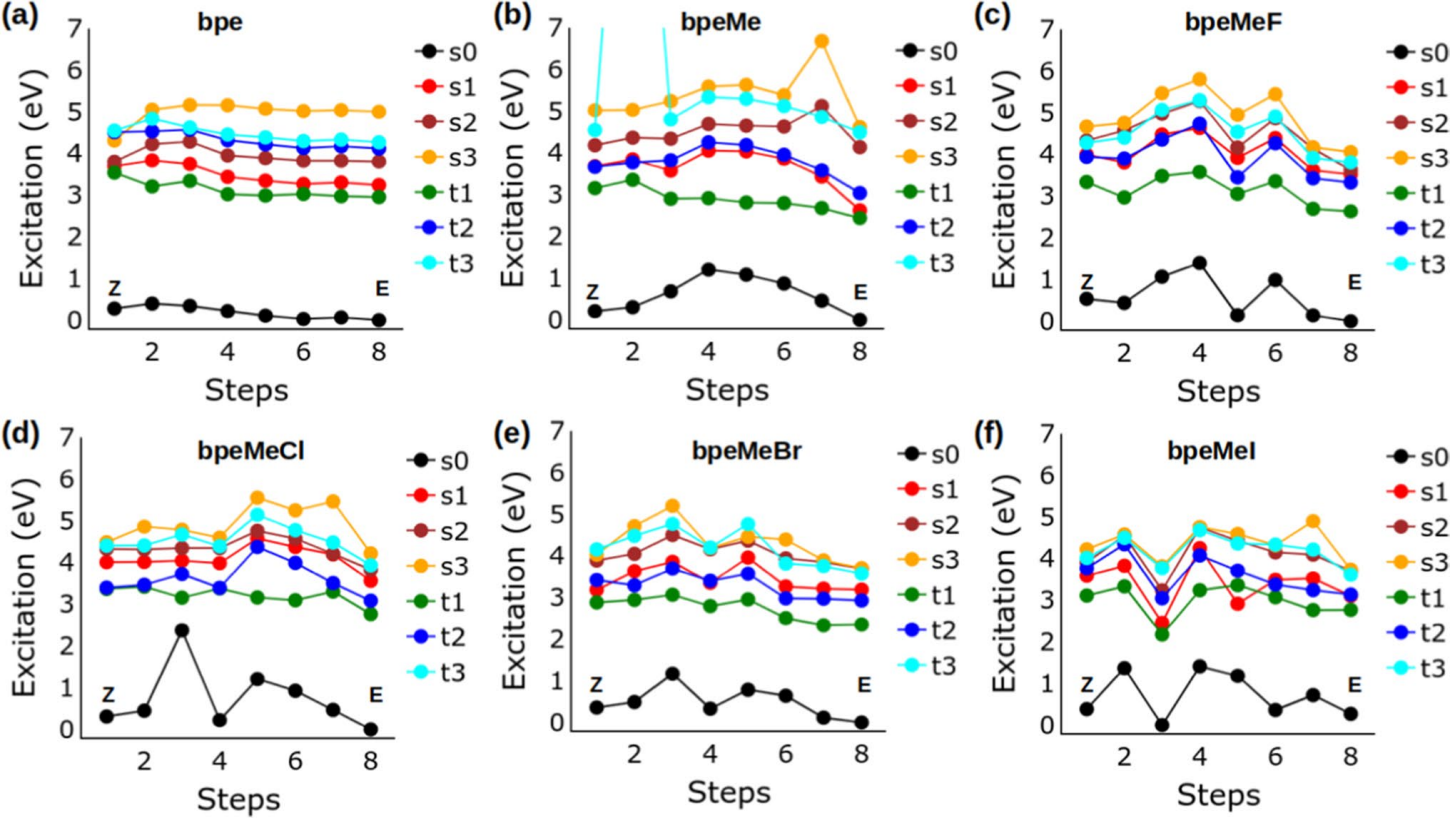
Excited state energies of (**a**) bpe, (**b**) bpeMe, (**c**) bpeMeF, (**d**) bpeMeCl, (**e**) bpeMeBr, and (**f**) bpeMeI, along the E → Z conformational change, using the NEVPT2 method (because of its high value the data point of bpeMe at step 2 in (b) was cut off)

The reaction path at the ground state and excited state for the E → Z isomerisation was studied using CASSCF and a more accurate multireference method, NEVPT2. Single point calculations, to determine CASSCF and NEVPT2 energies, were based on the ground state geometries of the reaction path identified in Fig. 2. The excited state reaction path using the NEVPT2 method is shown in Fig. 4, while that of the CASSCF method is shown in the Supplementary Information (Figure S1).

There is a significant difference between the excited state PES obtained using CASSCF and NEVPT2 methods. The s1 state was found above the t1 and t2 states in CASCF, with significant energy separation between s1 and t1 (above 1.0 eV), but when a more accurate NEVPT2 was used, s1 was found at a much lower energy difference from the ground state and in most cases overlapping with the t2 state, while being very close to t1. The s1 state was even found to overlap with t1 along the E → Z photoisomerisation PES of bpe, bpeMe, bpeMeBr and bpeMeI, when using NEVPT2. Another interesting feature of the excited state PES is found in bpeMeF and bpeMeCl, where there are many overlaps of the s1 and s2 states, indicating a strong possibility of internal conversion from the s2 excited state to the s1 state.

A very strong minimum was observed in both the GS and all the excited states of bpeMeI of the Z-isomer. Similar minima were also seen in bpeMeBr and bpeMeF, but mid-way between the E and Z isomers. BpeMeCl shows a unique PES at step 3; the GS energy went up but the t1 energy got lower, resulting in a very small energy gap between t1 and s0, which may lead to ISC. The PES of the t1 where the photoisomerization is expected to take place has a lower barrier compared to the ground state in all the molecules except bpeMeI, where the well that is found in s0 was also found in t1 in the Z-isomer zone. This may be a reason for the experimental quantum yield of the photoisomerization of bpeMeI being lower than that of bpeMeCl. Besides this, one would have expected more photoisomerization of bpeMeI, because of the significant level of overlapping of s1 and t1, which would enhance ISC.

**Fig. 5:**
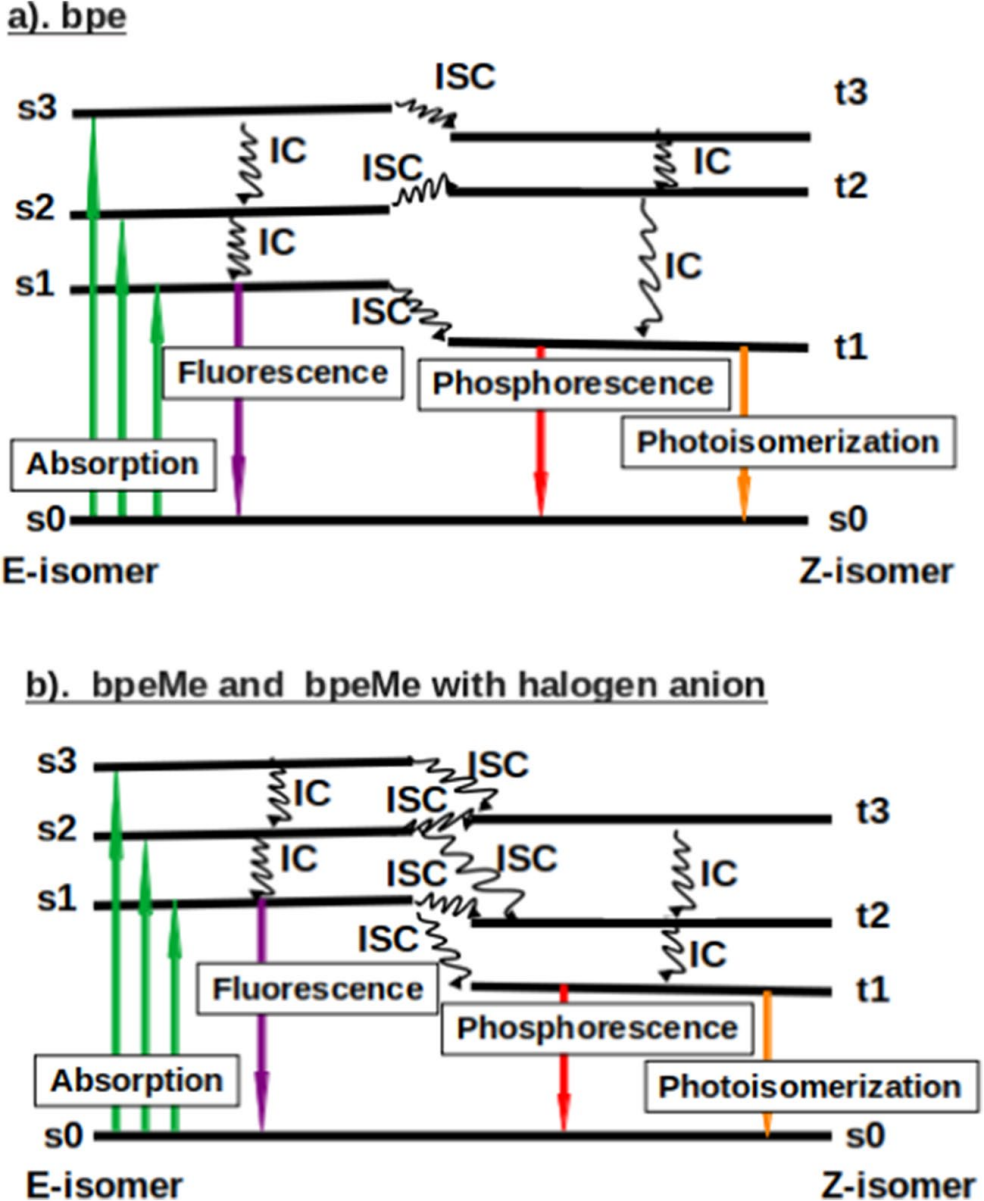
Schematic representation of the excited state energy levels (not drawn to scale) of (**a**) bpe, (**b**) bpeMe and its quaternary halogen salts (bpeMeF, bpeMeCl, bpeMeBr and bpeMeI), illustrating features related to the PES, obtained from the NEVPT2 method reported in Fig. 4

In the results obtained from NEVPT2, an overlap is seen between s1 and t1 in the E-isomers of bpe, bpeMe and bpeMeI, showing a very strong possibility of ISC from s1 to t1, which may lead to either photoisomerization or phosphorescence. The bpeMeI molecule shows two additional overlaps of s1 and t1 along the excited state PES. At step 5 the energy of s1 was found to be lower than that of t1. As for the Z isomer, only bpe and bpeMeBr show an overlap of the s1 and t1 states. All the molecules (except bpe) show several overlaps of t2 with s1. There is a unique behaviour of the t2 excited state in all the halogen salt molecules; the energies of t2 in many instances are found to be lower than that of s1, suggesting an ISC to the triplet state via t2, being an s1 → t2 → t1 photoisomerization mechanism. There is significant overlap t2 and t1 in bpeMeCl, especially near its Z isomer, which may be the reason for the experimental observation of increased photoisomerization in bpeMeCl as compared to bpeMeI [1]. The observed IC, ISC and possible photochemical and photophysical properties, as obtained from the results in Fig. 4, are summarised in Fig. 5.

### Singlet and triplet coupling with the Charge transfer of the molecules

The two major divisions of the excited states of organic molecular systems in terms of binding energies are:

1. strongly bound locally excited (LE) states, and.
2. weakly bound charge-transfer (CT) states [21, 22].

The weak binding energy of the CT can make use of both singlet and triplet excitons because of the possibility of a spin flip between the singlet and triplet exciton that results from spin mixing due to near-zero or negative energy splitting [21, 23].

This implies that where there is near-zero energy splitting between the singlet and triplet state, there is a high possibility of CT. Besides, the overlapping of the s1 and t2 states that is common to all the molecules besides bpe**,** additional overlap is observed between s1 and t1 in bpeMeI, resulting in a high level of CT, that was also experimentally observed in bpeMeI [1].

**Fig. 6:**
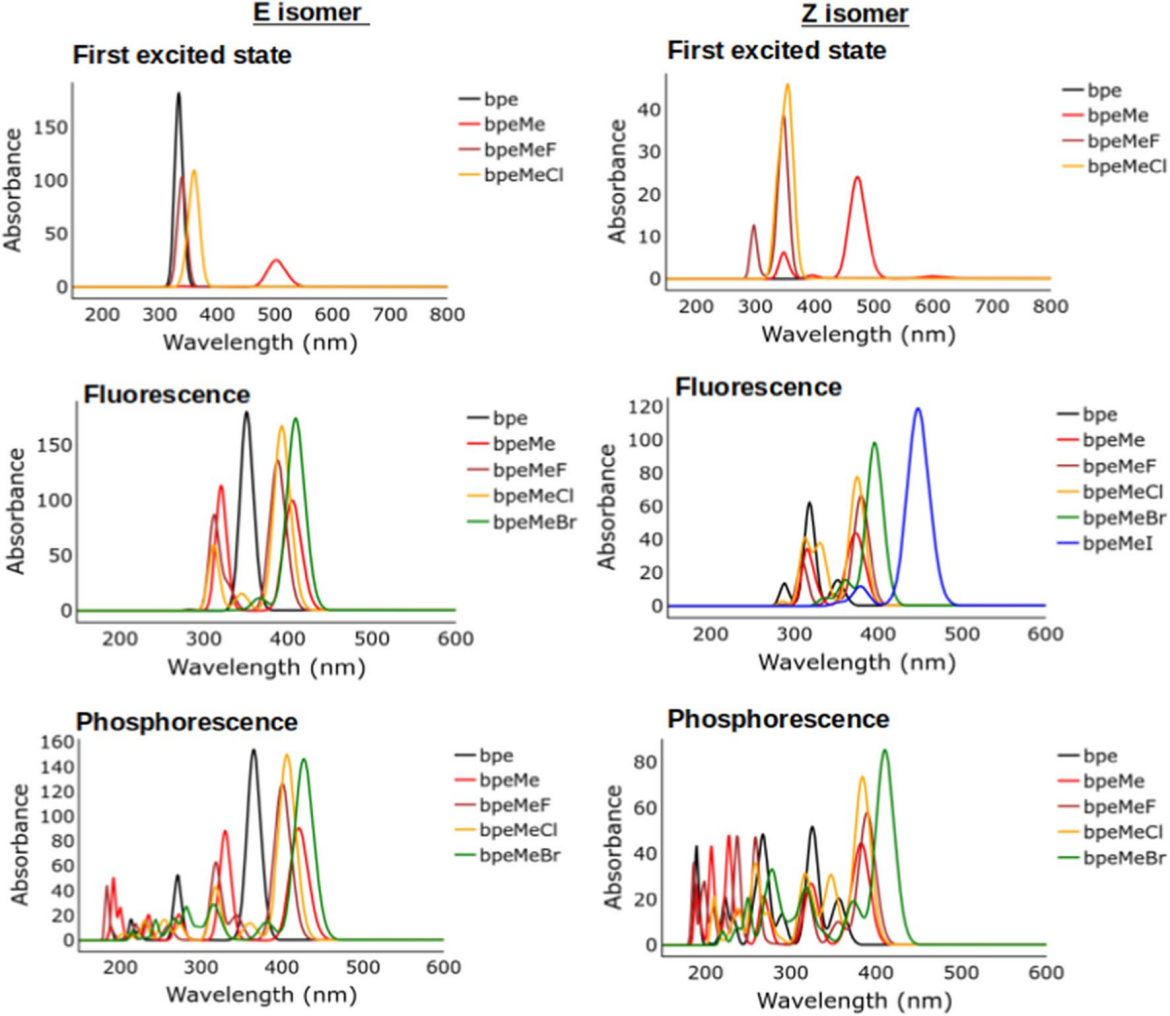
Absorption spectra of the first excited states of the molecules, as well as fluorescence and phosphorescence spectra. Due to higher atomic number, it was not possible to compute the first excited state spectra and some fluorescence and phosphorescence spectra of bpeMeBr and bpeMeI

The prominent absorption peaks that were found in the UV spectra of the spin–orbit coupling (SOC) calculation using the NEVPT2 method are shown in Table 1, while the UV spectra showing all the absorption peaks are represented in Fig. 6. There are some spin-forbidden excitations to the triplet state, which are shown in Table 1 as coming from the singlet ground state. However, the analysis of the excited state (Fig. 4) shows that they are not directly from the ground state but occur due to the overlapping of the singlet and triplet excited states in the molecules. The low level of charge transfer from the excited singlet state to the triplet state is obvious from the lower values of their frequencies (fosc in Table 1).

**Table 1:** Prominent absorption peaks from the SOC calculation using NEVPT2 with absorption arranged according to frequency strength, fosc, in each molecule. Excited state numbers and corresponding spin states are shown

Excitation	spin	Energy (cm^−1^)^a^	Wavelength (nm)	fosc^b^	T2 (D**2)^c^
Z-bpe					
0(s0) → 6(s3)^d^	0	32,532.2	307.4	4.49E-01	2.93E + 01
0(s0) → 4(s1)	0	27,587.4	362.5	3.18E-02	2.45E + 00
0(s0) → 5(s2)	0	28,427.3	351.8	2.21E-02	1.65E + 00
0(s0) → 1(t1)	1	26,322.0	379.9	8.70E-08	1.00E-05
0(s0) → 12(t3)	1	34,443.1	290.3	3.70E-08	0.00E + 00
E-bpe					
0(s0) → 5(s2)	0	30,728	325.4	1.09E + 00	7.53E + 01
0(s0) → 4(s1)	0	26,159.1	382.3	2.00E-09	0.00E + 00
0(s0) → 12(s3)	0	40,353.4	247.8	2.00E-09	0.00E + 00
Z-bpeMe					
0(s0) → 8(s2)	0	32,076.6	311.8	2.76E-01	1.83E + 01
0(s0) → 7(s1)	0	28,047.5	356.5	2.63E-01	1.99E + 01
0(s0) → 12(s3)	0	38,843.3	257.4	5.68E-02	3.10E + 00
0(s0) → 4(t2)	1	27,902.7	358.4	4.77E-06	3.60E-04
0(s0) → 11(t3)	1	35,009.4	285.6	2.90E-08	0.00E + 00
E-bpeMe					
0(s0) → 8(s2)	0	33,395.8	299.4	8.87E-01	5.64E + 01
0(s0) → 4(s1)	0	21,181.6	472.1	3.26E-01	3.27E + 01
0(s0) → 12(s3)	0	37,241.1	268.5	6.30E-02	3.59E + 00
Z-bpeMeF					
0(s0) → 7(s1)	0	27,920.0	358.2	3.35E-01	2.55E + 01
0(s0) → 12(s3)	0	33,299.1	300.3	2.14E-01	1.37E + 01
0(s0) → 11(s2)	0	30,618.3	326.6	7.43E-02	5.15E + 00
0(s0) → 4(t2)	1	27,482.7	363.9	1.68E-07	1.00E-05
0(s0) → 8(t3)	1	30,183.7	331.3	1.40E-08	0.00E + 00
E-bpeMeF					
0(s0) → 8(s2)	0	29,251.0	341.9	6.29E-01	4.57E + 01
0(s0) → 7(s1)	0	28,413.7	351.9	5.01E-01	3.75E + 01
0(s0) → 12(s3)	0	32,737.6	305.5	7.24E-02	4.69E + 00
0(s0) → 4(t2)	1	26,834.1	372.7	7.00E-09	0.00E + 00
0(s0) → 6(t2)	1	26,834.1	372.7	5.00E-09	0.00E + 00
Z-bpeMeCl					
0(s0) → 12(s3)	0	33,683.7	296.9	4.48E-01	2.83E + 01
0(s0) → 7(s1)	0	29,849.2	335.0	3.56E-01	2.53E + 01
0(s0) → 8(s2)	0	32,403.1	308.6	1.99E-01	1.31E + 01
0(s0) → 9(t3)	1	33,024.0	302.8	1.17E-06	8.00E-05
0(s0) → 11(t3)	1	33,024.0	302.8	6.67E-07	4.00E-05
E-bpeMeCl					
0(s0) → 7(s1)	0	28,730.0	348.1	6.87E-01	5.08E + 01
0(s0) → 12(s3)	0	34,027.5	293.9	6.33E-01	3.95E + 01
0(s0) → 8(s2)	0	30,886.6	323.8	5.27E-02	3.62E + 00
0(s0) → 4(t2)	1	24,889.9	401.8	6.00E-07	5.00E-05
0(s0) → 11(t3)	1	31,742.5	315.0	2.27E-07	2.00E-05
Z-bpeMeBr					
0(s0) → 4(s1)	0	22,788.1	438.8	4.98E-01	4.65E + 01
0(s0) → 8(s2)	0	28,480.3	351.1	1.91E-01	1.43E + 01
0(s0) → 9(s3)	0	29,653.7	337.2	6.84E-04	4.90E-02
0(s0) → 7(t2)	1	24,698.3	404.9	1.11E-04	9.57E-03
0(s0) → 5(t2)	1	24,696.9	404.9	6.19E-05	5.32E-03
E-bpeMeBr					
0(s0) → 7(s1)	0(96.78%),1(3.05%)^e^	25,640.7	390	1.11E + 00	9.22E + 01
0(s0) → 10(t3)	1(96.60%),0(3.14%%)	28,972.2	345.2	4.16E-02	3.05E + 00
0(s0) → 11(s2)	0(94.68%),0(4.21%)	29,920.8	334.2	1.34E-03	9.54E-02
0(s0) → 6(t2)	1	23,621.4	423.3	6.91E-04	6.21E-02
0(s0) → 12(s3)	1,1,0,0(67.53)	30,677.6	326.0	3.58E-04	2.48E-02
Z-bpeMeI					
0(s0) → 4(s1)	0	25,844.3	386.9	6.85E-01	5.63E + 01
0(s0) → 8(s2)	0	28,445.3	351.6	9.81E-04	7.33E-02
0(s0) → 12(s3)	0	30,951.5	323.1	9.42E-04	6.46E-02
0(s0) → 5(t2)	1	27,280.8	366.6	2.58E-07	2.00E-05
0(s0) → 11(t3)	1	29,285.4	341.5	1.90E-08	0.00E + 00
E-bpeMeI					
0(s0) → 4(s1)	0	22,858.2	437.5	9.09E-01	8.44E + 01
0(s0) → 12(s3)	0	27,935.4	358.0	1.33E-03	1.01E-01
0(s0) → 11(s2)	0	27,709.8	360.9	6.43E-04	4.93E-02
0(s0) → 7(t2)	1	23,106.2	432.8	1.23E-05	1.13E-03
0(s0) → 10(t3)	1	26,960.8	370.9	9.59E-06	7.60E-04

^a^1 nm (wavelength) = 10 000 000/cm^−1^ (Energy)

Thus as example, Energy (3 2532.2 cm^−1^) = 10 000 000/32 532.2 nm = 307.39 nm

^b^fosc = frequency of the excitation

^c^T2 (D**2) is the square of the electric transition dipole moments (T) in Debye^2^ that is directly proportional to the fosc in μs^−1^

^d^The notation 0(s0) → 6(s3) indicates an excitation from the ground state (s0) to the third excited state (s3); 0 indicates the ground state and 6 stands for the sixth energy level

^e^The notation 0(s0) → 7(s1), s(96.78%), t(3.05%) implies excitation from the ground state 0(s0) to first singlet excited state 7(s1), characterised to have orbital mixing of 96.78% singlet (s(96.78%)) and 3.05% triplet (t(3.05%)

The E-bpeMeBr molecules have the highest level of charge transfer to the triplet excited state (0(s0) → 10(t3) fosc = 4.16E-02), with two instances of singlet and triplet state mixing, as indicated in Table 1 by “0(s0) → 7(s1) s(96.78%),t(3.05%)” and “0(s0) → 10(t3) t(96.60%),s(3.14%%)”. The notation “0(s0) → 7(s1) s(96.78%),t(3.05%)” implies excitation from the ground state 0(s0) to first singlet excited state 7(s1), characterised to have orbital mixing of 96.78% singlet (s(96.78%)) and 3.05% triplet (t(3.05%). The strongest charge transfer to the triplet state was found in E-bpeMeBr which is the consequence of overlapping of t2 with s1 excited states (Fig. 4). E-bpeMeI (as can be seen in Table 1) also shows significant charge transfer from the singlet excited state to the triplet state (0(s0) → 7(t2) and 0(s0) → 10(t3)) that are spin forbidden. The origin of this charge transfer in E-bpeMeI to t2 and t3 is from the overlapping of s1 with t2 and the overlapping of t3 with both s2 and s3 (Fig. 4).

The UV spectra obtained for the E-isomer and Z-isomers for each of the molecules are shown in Fig. 6. There is a stronger absorption peak in the E-isomers compared to their Z-isomers. In the E-isomer, bpe has the strongest peak, but bpeMe with halogen anions is stronger in Z-isomers, especially that of bpeMeCl and bpeMeBr. Phosphorescence is dominated by many peaks because of many possible ISCs in the systems, as shown in Figs. 4 and 5. The fluorescence and phosphorescence spectra show absorption peaks at much longer wavelengths towards the visible region, in order of bpeMeBr > bpeMe, bpeMe(F, Cl) > bpe. This shows that bpeMeBr with the stronger electron-donating anion Br^-^ (relative to F^-^) undergo fluorescence and phosphorescence at much lower energies, while molecules with strong electron-withdrawing (weak electron-donating) anions like bpeMeF and bpeMeCl, appear at a slightly higher energy level than that of the bpeMe cation.

### Fluorescence and Phosphorescence properties

Fluorescence and phosphorescence of the E and Z isomers of the molecules were computed as shown in Table 2. Phosphorescence values were computed as the average of the three triplet substrates, as reported in the literature [24]:

**Table 2:** Computed fluorescence and phosphorescence rate constant (s^−1^) values of the E and Z isomers. Percentage vibronic couplings in forbidden transitions (the so-called Herzberg-Teller effect, HT) and Frank–Condon (FC) values are listed

Mol^a^	FLUOR (s^−1^)	FC(%)	HT(%)
E-bpe	2.92E + 10	0	100
Z-bpe	6.83E + 07	79.96	20.04
E-bpeMe	2.96E + 08	103.89	-3.89
Z-bpeMe	3.63E + 08	22.44	77.56
E-bpeMeF	5.97E + 08	85.75	14.25
Z-bpeMeF	2.08E + 08	102.99	-2.99
E-bpeMeCl	4.78E + 08	89.34	10.66
Z-bpeMeCl	2.58E + 08	70.23	29.77
E-bpeMeBr	1.12E + 08	90.14	9.86
Z-bpeMeBr	8.99E + 06	96.39	3.61
**Mol**	**PHOSPH(s**^**−1**^**)**	**FC(%)**	**HT(%)**
E-bpe	1.36E-01	13.01	86.99
Z-bpe	1.16E + 00	20.17	79.83
E-bpeMe	8.26E-04	0.71	99.29
Z-bpeMe	2.52E-03	15.48	84.52
E-bpeMeF	1.01E + 01	0.33	99.67
Z-bpeMeF	3.55E + 00	6.17	93.83
E-bpeMeCl	4.65E + 00	1.31	98.69
Z-bpeMeCl	2.40E + 00	2.71	97.29
E-bpeMeBr	3.02E + 02	27.02	72.98
Z-bpeMeBr	1.35E + 02	24.36	75.64

aThe atomic mass of bpeMeI is too high and could not be computed.

$${k}_{phosph}= \frac{{k}_{1}+ {k}_{2}+ {k}_{3}}{3}$$

In molecules with heavy atoms, the vibronic coupling that is associated with a large Herzberg–Teller (HT) contribution may be negligible, because the spin–orbit coupling matrix elements (SOCME) are expected to be relatively large, i.e. > 5 cm^−1^.

Computed fluorescence and phosphorescence rate constant values of the molecules are shown in Table 2. All the molecules give very high fluorescence constant values compared to their phosphorescence constant values. The molecule bpeMeCl with the weak electron-donating Cl^-^ anion shows increased photoisomerization and fluorescence, while derivatives with highly electron-donating anions like I^−^ are dominated by electron transfers that compete with both its fluorescence and photoisomerization mechanisms [1]. Our results support this observation, as the molecules bpeMeF and bpeMeCl showed a higher fluorescence constant compared to bpeMeBr. Fluorescence is mainly from the FC with less contribution of HT, except for the E-bpe and Z-bpeMe. Phosphorescence in turn is mainly from HT, which implies phosphorescence resulting more from vibronic coupling. This implies that transitions between the excited and ground states are not purely electronic transitions, but involve intramolecular interactions between electrons and vibrations from the motions of nuclei [25]. However, the contribution of FC to the phosphorescence constant is more significant in molecule bpeMeBr than the rest, which possibly leads to higher phosphorescence of bpeMeBr, compared to the rest of the molecules.

### Molecular property changes in the E to Z isomers

**Fig. 7:**
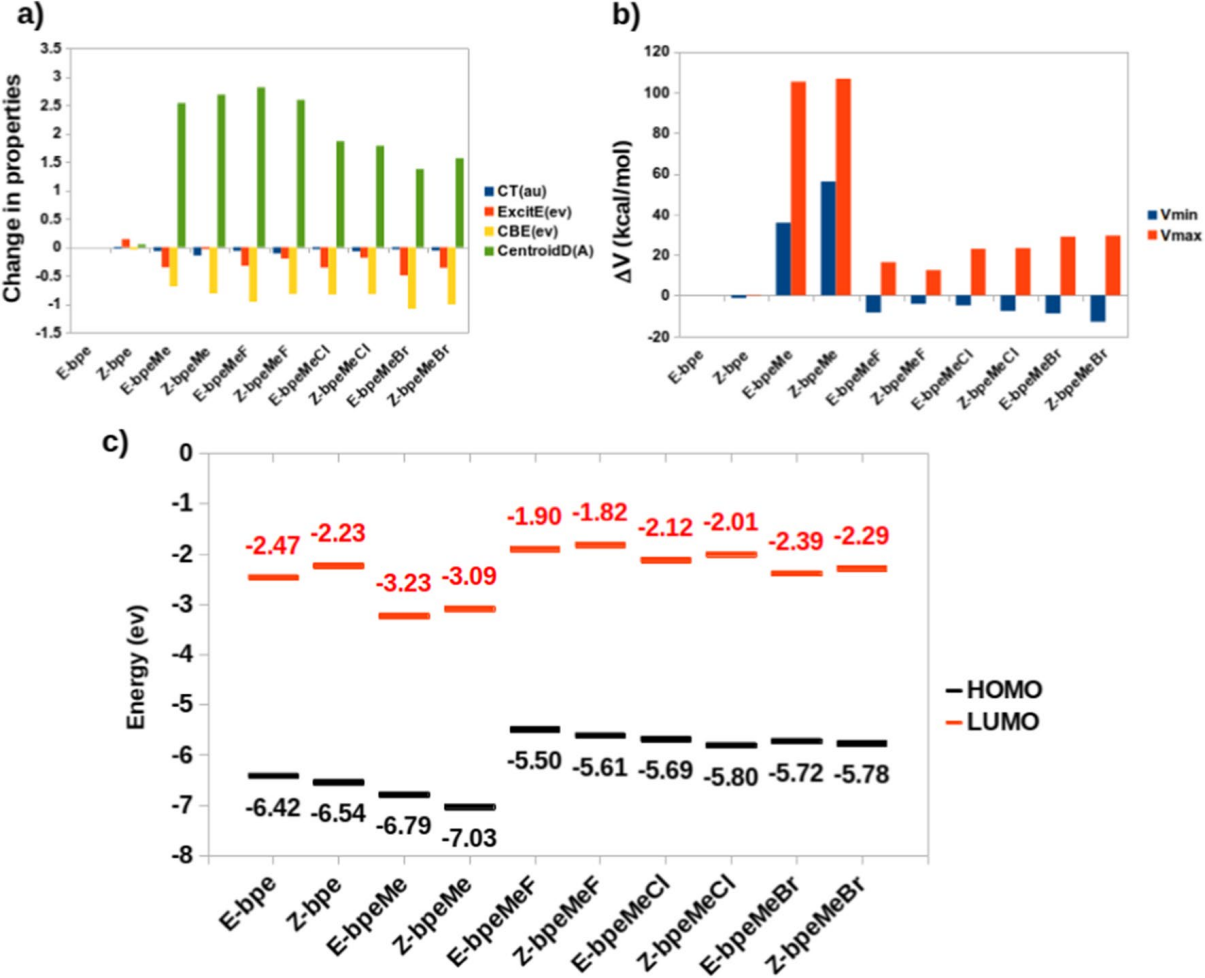
(**a**) Changes in the computed charge transfer (CT), excitation energy (ExcitE), Columbic energy (CBE), and centroid distance between the hole and electron of each of the molecules from that of Ebpe. (**b**) Changes in values of V_min_ and V_max_ of each of the ground states of the molecules from that of Ebpe. (**c**) HOMO and LUMO energies of the ground states of the molecules

Some of the properties that were computed for comparison of E and Z isomers include charge transfer, excitation energy, Coulomb attractive energy and centroid distance between the hole and electron points on each of the molecules obtained from TDDFT (NStates = 25) and their V_min_ and V_max_ values obtained from MESP analyses. The critical points (CPs) of MESP were located by topographical analysis. A (3, + 3) CP is represented by a MESP minimum (V_min_); a maximum is denoted by (3, -3) and saddle points by (3, + 1) and (3, -1) [26, 27]. These values are listed under Supplementary Information, Tables S2 and S3. Changes in computed properties, namely charge transfer (CT), excitation energy (ExcitE), Columbic energy (CBE), centroid distance between the hole and electron, MESP minimum V_min_ and maximum V_max_, of other molecules from that of the E-bpe, are shown in Fig. 7.

Among the computed properties, a significant change in centroid distance was observed in all molecules when compared to that of E-bpe. The centroid distance in bpeMeBr is a little smaller than in bpeMe and the halogenated compounds. The bpeMeBr is also characterised by lower values of Coulomb attractive energy and excitation energy. In all the molecules with halogens the excitation and Coulomb attractive energies are lower in the E-isomers than in the Z-isomers.

As shown in Fig. 7, there is an observed increase in the bandgap between the HOMO and LUMO for all the Z-isomers compared to the E-isomers. The reason for the increase in the bandgap of the Z-isomers is due to the increase in its LUMO and decrease in its HOMO energies. The HOMO and the LUMO of bpeMe decrease significantly because of the presence of the methyl group as compared to bpe. In the presence of halogen anions, the HOMO and LUMO energies increased when compared to bpe. The bpeMeBr molecule has the lowest bandgap (E-isomer = 3.34, Z-isomer = 3.49) among all the molecules while bpe has the highest bandgap (E-isomer = 3.95, Z-isomer = 4.31).

**Fig. 8:**
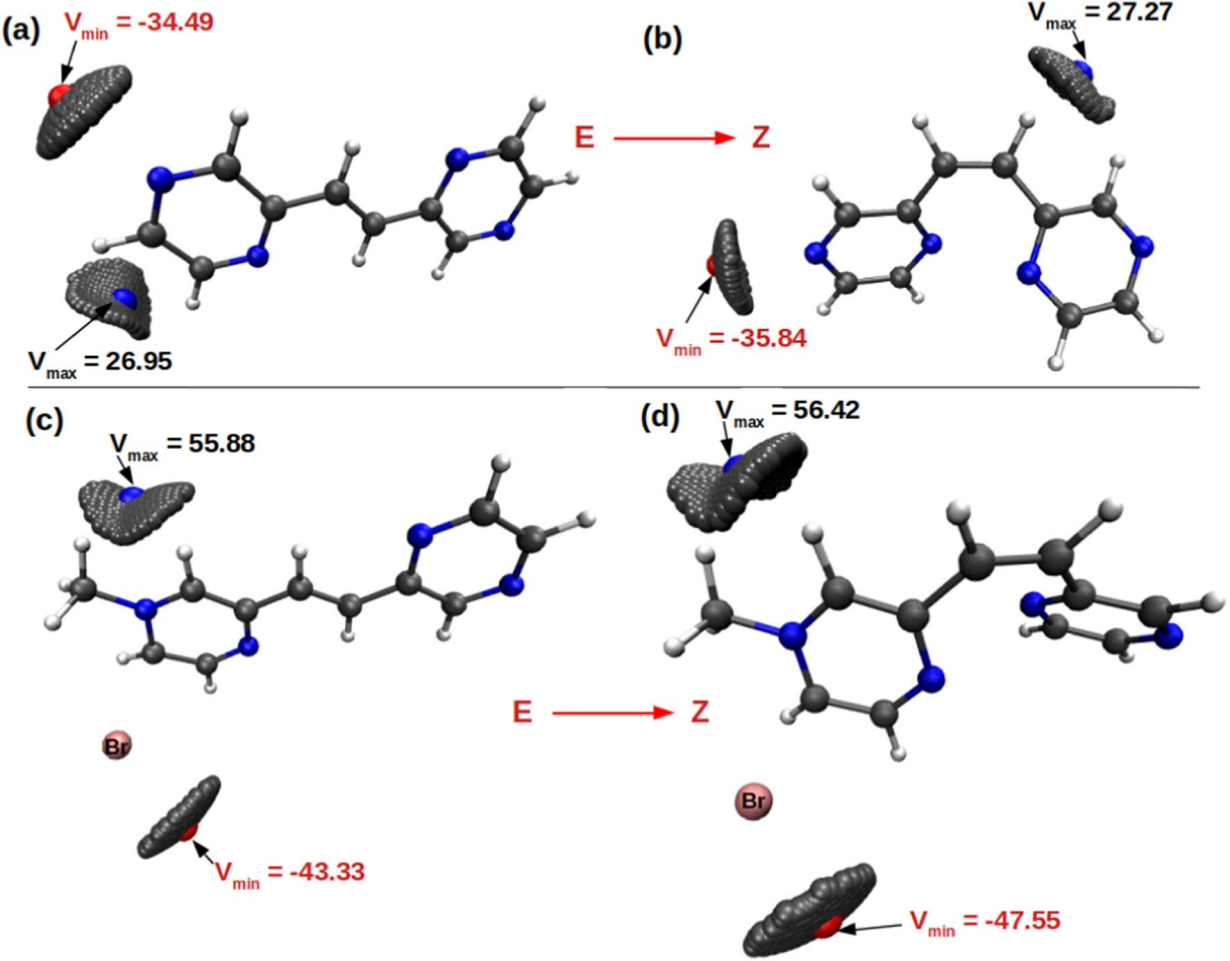
MESP analyses, showing V_min_ and V_max_ locations, for (**a**) E-bpe, (**b**) Z-bpe, (**c**) E-bpeMeBr and (**d**) Z-bpeMeBr

MESP minima (V_min_) increase in magnitude in the molecules with halogen anions, from F^−^ to Br^−^. In the presence of halogens, the V_min_ and V_max_ values drop significantly, especially in the presence of F ^−^, when compared to Br^−^. V_min_ decreases while V_max_ increases on going from the E to the Z isomers. V_min_ and V_max_ locations for bpe and bpeMeBr are shown in Fig. 8. V_min_ is located near the halogen, in both the E and Z isomers. However, in unsubstituted bpe, there is a change in the V_max_ position on going from the E to the Z isomer, the V_min_ position however remains in the same location in both isomers.

## Conclusion

The results obtained from the excited state study of the derivatives of bpe, bpeMe and the quaternary bpeMe halogen salts are summarised as follows:

- The excited state structures of molecules with Cl^−^, Br^−^, and I^−^ anions show a more significant change in the t1 structure than s1 structures when superimposed on the GS structure. On the contrary, the excited state of bpeMeF, bpe and bpeMe show a higher RMSD of the s1 compared to t1.
- The excited state PES of bpeMeI suggests a higher level of ISC because of the significant level of overlap between s1 and t1, while in bpeMeF and bpeMeCl more overlap of s1 and s2 can result in IC from s2 to s1.
- The PES of t1 where the photoisomerization is expected to take place has a lower barrier compared to the ground state in all the molecules except for bpeMeI, where a well that is found in s0 is also in t1 at the zone of the Z isomer. This may be a reason for the experimental quantum yield of bpeMeI photoisomerization being found lower than that of bpeMeCl.
- A lower s1 energy than t1 energy was observed along the excited state PES of bpeMeI, further explaining why the experimental photoisomerization for bpeMeI was lower than that for bpeMeCl. Photoisomerism is expected to take place at a point along PES where t1 is at a higher energy value compared to s1. Another reason for the observed higher photoisomerization in bpeMeCl compared to bpeMeI may be traced to the overlapping of t2 and t1 near the Z isomer of bpeMeCl.
- A near-zero splitting between s1 and t1 is common along the excited state PES of bpeMeI, which may be responsible for higher experimentally reported CT in bpeMeI. This supports the view that near-zero or negative energy splitting leads to the possibility of a spin flip, which thereby enhances CT [21, 23].
- Molecules with halogens are predicted to undergo ISC to the triplet state via t2 which is highly overlapped with s1 and can initiate a possible s1 → t2 → t1 photoisomerization mechanism.
- Fluorescence and phosphorescence show absorption peaks to appear at a much longer wavelength towards the visible region, and the following order: λ = bpeMeBr > bpeMe, bpeMe(F, Cl) > bpe. This shows that the bpeMe cation, compared to the neutral bpeMeBr, can undergo fluorescence and phosphorescence at much lower energies. The molecules with weak electron-donating anions like bpeMeF and bpeMeCl appear at somewhat higher energy than that of the bpeMe.

### Supplementary Information

Below is the link to the electronic supplementary material.

Supplementary file1 (PDF 439 KB)

## Data Availability

Data available in article and the Supplementary Information.

## References

[1] Shim SC, Kim MS (1988). Photochemical E → Z isomerization of quaternary salts of E-1,2-bispyrazinylethylene. J Photochem Photobiol A Chem.

[2] Lower SK, El-Sayed MA (1966). The Triplet State and Molecular Electronic Processes in Organic Molecules. Chem Rev.

[3] Safiarian MS, Ugboya A, Khan I, Marichev KO, Grant KB (2023). New Insights into the Phototoxicity of Anthracene-Based Chromophores: The Chloride Salt Effect†. Chem Res Toxicol.

[4] Saxena B, Patel RI, Sharma A (2023). Recent Advances in Electron Donor-Acceptor (EDA)-Complex Reactions involving Quaternary Pyridinium Derivatives. Adv Synth Catal.

[5] Tang Z, Han H, Ding J, Zhou P (2021). Dual fluorescence of 2-(2′-hydroxyphenyl) benzoxazole derivativesviathe branched decays from the upper excited-state. Phys Chem Chem Phys.

[6] Tang Z, Li P, Liu J, Zhou P (2020). Carbonyl stretch as a Franck−Condon active mode and driving force for excited-state decay of 8-methoxy-4-methyl-2H-benzo[g]chromen-2-one from nπ* State. J Phys Chem B.

[7] Frisch MJ, Trucks GW, Schlegel HB, Scuseria GE, Robb MA, Cheeseman JR, Scalmani G, Barone V, Petersson GA, Nakatsuji H, Li X, Caricato M, Marenich AV, Bloino J, Janesko BG, Gomperts R, Mennucci B, Hratchian HP, Ortiz JV, Izmaylov AF, Sonnenberg JL, Williams-Young D, Ding F, Lipparini F, Egidi F, Goings J, Peng B, Petrone A, Henderson T, Ranasinghe D, Zakrzewski VG, Gao J, Rega N, Zheng G, Liang W, Hada M, Ehara M, Toyota K, Fukuda R, Hasegawa J, Ishida M, Nakajima T, Honda Y, Kitao O, Nakai H, Vreven T, Throssell K, Montgomery J, Peralta JE, Ogliaro F, Bearpark MJ, Heyd JJ, Brothers EN, Kudin KN, Staroverov VN, Keith TA, Kobayashi R, Normand J, Raghavachari K, Rendell AP, Burant JC, Iyengar SS, Tomasi J, Cossi M, Millam JM, Klene M, Adamo C, Cammi R, Ochterski JW, Martin RL, Morokuma K, Farkas O, Foresman JB, DJ Fox (2016) Gaussian 16, Revision B.01. Gaussian, Inc., Wallingford CT

[8] Henkelman G, Jónsson H (2000). Improved tangent estimate in the nudged elastic band method for finding minimum energy paths and saddle points. J Chem Phys.

[9] Mills G, Jónsson H, Schenter GK (1995). Reversible work transition state theory: application to dissociative adsorption of hydrogen. Surf Sci.

[10] F. Neese, Software update: the ORCA program system, version 4.0, Wiley Interdiscip. Rev. Comput. Mol. Sci. 8 (2018). 10.1002/wcms.1327.

[11] Neese F (2012). The ORCA program system. Wiley Interdiscip Rev Comput Mol Sci.

[12] Angeli C, Cimiraglia R, Evangelisti S, Leininger T, Malrieu J-P (2001). Introduction of n-electron valence states for multireference perturbation theory. J Chem Phys.

[13] Angeli C, Cimiraglia R, Malrieu J-P (2002). n-electron valence state perturbation theory: A spinless formulation and an efficient implementation of the strongly contracted and of the partially contracted variants. J Chem Phys.

[14] Weigend F, Ahlrichs R (2005). Balanced basis sets of split valence, triple zeta valence and quadruple zeta valence quality for H to Rn: Design and assessment of accuracy. Phys Chem Chem Phys.

[15] Marenich AV, Cramer CJ, Truhlar DG (2009). Universal Solvation Model Based on Solute Electron Density and on a Continuum Model of the Solvent Defined by the Bulk Dielectric Constant and Atomic Surface Tensions. J Phys Chem B.

[16] Humphrey W, Dalke A, Schulten K (1996) VMD: visual molecular dynamics. J Mol Graph 14:33–38. 10.1016/0263-7855(96)00018-5 10.1016/0263-7855(96)00018-5 8744570

[17] Lu T, Chen F (2012). Multiwfn: a multifunctional wavefunction analyzer. J Comput Chem.

[18] Lu T, Chen F (2012). Quantitative analysis of molecular surface based on improved Marching Tetrahedra algorithm. J Mol Graph Model.

[19] Shiri F, Pirhadi S, Ghasemi JB (2015). Alignment independent 3D-QSAR, quantum calculations and molecular docking of Mer specific tyrosine kinase inhibitors as anticancer drugs. Saudi Pharm J.

[20] F.J. Luque, J.M. López, M. Orozco, Perspective on “Electrostatic interactions of a solute with a continuum. A direct utilization of ab initio molecular potentials for the prevision of solvent effects,” Theor. Chem. Accounts Theory, Comput. Model. (Theoretica Chim. Acta). 103 (2000) 343–345. 10.1007/s002149900013.

[21] Li W, Pan Y, Yao L, Liu H, Zhang S, Wang C, Shen F, Lu P, Yang B, Ma Y (2014). A Hybridized Local and Charge-Transfer Excited State for Highly Efficient Fluorescent OLEDs: Molecular Design, Spectral Character, and Full Exciton Utilization. Adv Opt Mater.

[22] Grabowski ZR, Rotkiewicz K, Rettig W (2003). Structural Changes Accompanying Intramolecular Electron Transfer: Focus on Twisted Intramolecular Charge-Transfer States and Structures. Chem Rev.

[23] Barford W, Bursill RJ, Makhov DV (2010). Spin-orbit interactions between interchain excitations in conjugated polymers. Phys Rev B.

[24] V.A. Online, K. Mori, T.P.M. Goumans, E. Van Lenthe, F. Wang, Predicting phosphorescent lifetimes and zero-field splitting of organometallic complexes with time-dependent density functional theory, (2014) 14523–14530. 10.1039/c3cp55438d 10.1039/c3cp55438d 24664116

[25] F.F. Kong, X.J. Tian, Y. Zhang, Y.J. Yu, S.H. Jing, Y. Zhang, G.J. Tian, Y. Luo, J.L. Yang, Z.C. Dong, J.G. Hou, Probing intramolecular vibronic coupling through vibronic-state imaging, Nat. Commun. 12 (2021). 10.1038/s41467-021-21571-z 10.1038/s41467-021-21571-z PMC7904785 33627671

[26] B.A. Anjali, C.H. Suresh, Electronic effect of ligands: Vs. reduction potentials of Fischer carbene complexes of chromium: A molecular electrostatic potential analysis, New J. Chem. 42 (2018) 18217–18224. 10.1039/c8nj04184a.

[27] Gadre SR, Shirsat RN (2000) Electrostatics of atoms and molecules. Universities Press, India

