# Supplementary material for: Theoretical study of the photoisomerization of 1,2-bispyrazinyl-ethylene and the halogen ion salts of 1-Pyrazinyl-2-(4’-methylpyrazinyl)ethylene
Source: J Mol Model. 2024 Mar 20;30(4):109. doi: 10.1007/s00894-024-05881-9 (PMC10954876; doi:10.1007/s00894-024-05881-9)
Supplement: Supplementary file 1 — Supplementary file1 (PDF 439 KB) [file 894_2024_5881_MOESM1_ESM.pdf]

Theoretical Study of the Photoisomerization of 1,2-Bispyrazinyl-Ethylene and the Halogen Ion Salts of 1-Pyrazinyl-2-(4'-Methylpyrazinyl)ethylene

Adebayo A. Adeniyi*[a,b], Jeanet Conradie[a], Karel G. von Eschwege[a]

[a] Department of Chemistry, University of the Free State, PO Box 339, Bloemfontein, 9300, South Africa

[b] Department of Industrial Chemistry, Federal University Oye-Ekiti, Nigeria

* Corresponding author: Adebayo A. Adeniyi, email: adeniyiaa@ufs.ac.za, Azeez.adebayo.adeniyi2@gmail.com

Supporting information

Table of Contents

Supporting information 1
- Figures 2
- Tables 3
- Optimized geometries 4
  - i). Z-bpe 4
  - ii). E-bpe 4
  - iii). Z-bpeMeBr 5
  - iv). E-bpeMeBr 5
  - v). Z-bpeMeCl 6
  - vi). E-bpeMeCl 6
  - vii). Z-bpeMeF 7
  - viii). E-bpeMeF 7
  - ix). Z-bpeMe 8
  - x). E-bpeMe 8

Figures

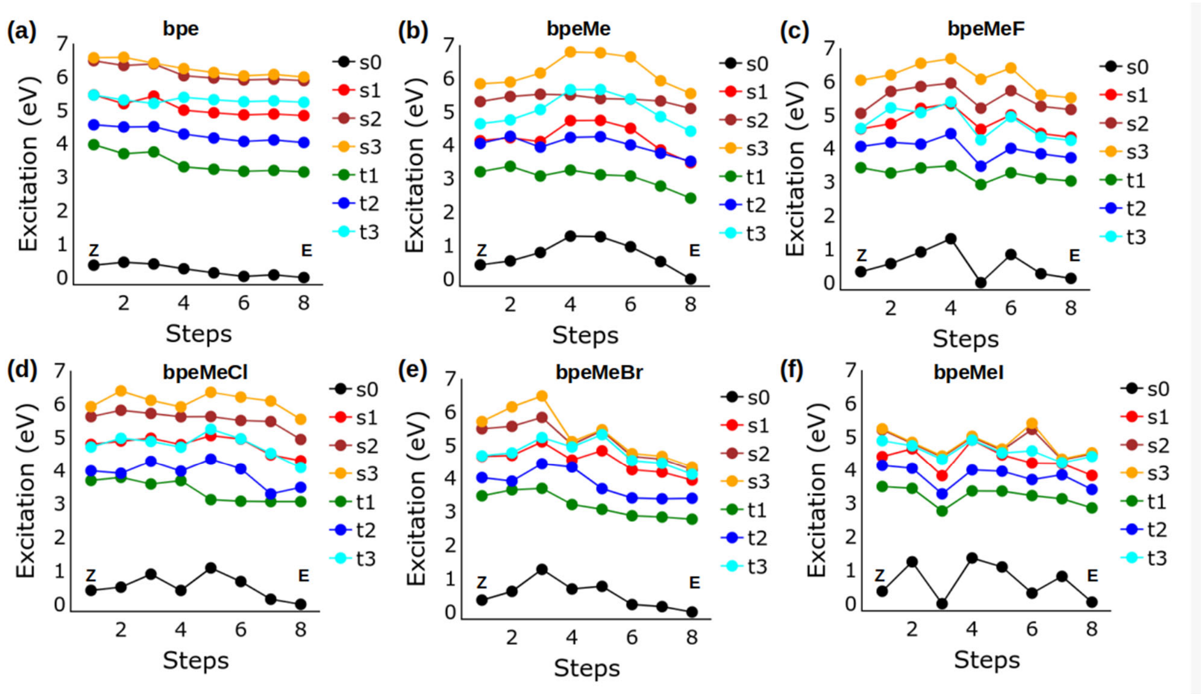

Figure S1: The excited state energy features of (a) bpe, (b) bpeMe, (c) bpeMeF, (d) bpeMeCl, (e) bpeMeBr, (f) bpeMeI along the E → Z conformational change using CASSCF method

Tables

Table S1: The computed charge transfer (CT), Excitation Energy (ExcitE), Columbic Energy (CBE) and Centroid distance between the hole and electron of each of the molecules in their E and Z isomers

Mol	CT(au)	ExcitE(ev)	CBE(ev)	CentroidD(A)
E-bpe	0.573	3.355	5.412	0.000
Z-bpe	0.543	3.497	5.366	0.050
E-bpeMe	0.500	2.999	4.719	2.535
Z-bpeMe	0.423	3.317	4.596	2.682
E-bpeF	0.504	3.026	4.450	2.807
Z-bpeF	0.458	3.152	4.586	2.589
E-bpeCl	0.532	2.995	4.578	1.864
Z-bpeCl	0.496	3.165	4.585	1.784
E-bpeBr	0.530	**2.859**	**4.330**	**1.373**
Z-bpeBr	0.509	**2.990**	**4.405**	**1.562**

Table S2: The values of the MESP minimum (Vmin) and maximum (Vmax) for each of the molecules in their E and Z isomers

Mol	Vmin	Vmax
E-bpe	-34.49	26.95
Z-bpe	-35.84	27.27
E-bpeMe	1.24	132.09
Z-bpeMe	21.58	133.71
E-bpeF	-42.88	43.26
Z-bpeF	-38.66	39.37
E-bpeCl	-39.41	49.77
Z-bpeCl	-42.13	50.25
E-bpeBr	**-43.33**	**55.88**
Z-bpeBr	**-47.55**	**56.42**

Optimized geometries

i). Z-bpe

C	-1.60088620598878	0.67193317883421	0.06587715149248
N	-1.30168696038076	-0.37174862877668	-0.71820699820454
C	-2.20516092308139	-1.33888179422048	-0.80629262410897
H	-1.96545684342633	-2.18166797972452	-1.44705224881181
C	-3.41784890037636	-1.28628532215673	-0.12397284476014
H	-4.14234452340880	-2.08948807412009	-0.20473808449345
N	-3.73976611290713	-0.24492414399056	0.64053057253726
C	-2.83855088360186	0.72709345317015	0.72326794824192
H	-3.09639718467643	1.58542412588352	1.33618355163802
C	-0.66631335703802	1.79841657655677	0.18191461767842
H	-1.14778361959251	2.76111264397601	0.32542110357130
C	0.67142862696140	1.80408813938587	0.10571547762649
H	1.15311463014750	2.77738838515076	0.10732056263369
C	1.60496535648996	0.67148666646204	0.04594963659023
C	2.81583435107026	0.80570465118021	-0.64835623709747
H	3.05562871950669	1.73333346748886	-1.15910264995630
N	3.71144149800856	-0.17201279971121	-0.72551293752423
C	3.41183005652667	-1.29982005491959	-0.08441907780027
H	4.13182957994209	-2.10950292343680	-0.13527053825595
C	2.22829901371215	-1.43315091975514	0.63665345261652
H	2.00869707230796	-2.34825633731033	1.17763860720108
N	1.32912760980515	-0.46024230996630	0.70645355918573

ii). E-bpe

C	-1.91797602849769	-0.00071380342631	0.03208720997935
N	-2.24252313197249	0.08916557835454	-1.26810539563054
C	-3.53226364850636	0.11059766681505	-1.57008258636163
H	-3.79402641792005	0.18311740054215	-2.62109522013312
C	-4.52460548993852	0.04369169014413	-0.59322215321103
H	-5.57559155259324	0.06240574627973	-0.86020049981651
N	-4.22077303107340	-0.04550910984912	0.69966152592185
C	-2.92830143617565	-0.06709754560546	1.00406566635017
H	-2.67029380557217	-0.13981659057507	2.05600116207336
C	-0.51441669748010	-0.02893601221573	0.42839724754742
H	-0.30806771665128	-0.10213691120397	1.49027661858343
C	0.51441192238667	0.02910370631886	-0.42837393322186
H	0.30811794305350	0.10230922470024	-1.49026323224850
C	1.91796505019277	0.00077873303697	-0.03205348074521
C	2.92832514932613	0.06707119702177	-1.00406955408761
H	2.67015087588328	0.13978291733450	-2.05597011226102
N	4.22074487783556	0.04540180121654	-0.69968914926333
C	4.52462020929976	-0.04379358460558	0.59321124930009
H	5.57560155453805	-0.06256844130311	0.86018826742122
C	3.53229461435081	-0.11061490371883	1.57006038560086
H	3.79408016392748	-0.18314235661178	2.62106747465342
N	2.24252859558695	-0.08909640264952	1.26810850954919

iii). Z-bpeMeBr

C	0.63416514077650	0.74931043495873	-0.06017095514827
N	0.29624068358537	-0.35613661815110	-0.80284132842160
C	1.20355600246323	-1.25509885500576	-1.01184174034151
H	0.95195394617187	-2.10781495926303	-1.63219674279733
C	2.54013918712686	-1.16783758921185	-0.50833778211811
H	3.17257033210737	-2.03667517714052	-0.45018469609459
N	2.77652292730680	-0.17528739573021	0.38640296283510
C	1.87579392169322	0.83978959733451	0.51141690544716
H	2.18012236702902	1.68005341255468	1.11802045497650
C	-0.31673353552115	1.84798723347389	0.09269491088921
H	0.14690065362344	2.82175300249064	0.21667090869087
C	-1.65811429836660	1.82690745851438	0.05528864128325
H	-2.15466646176027	2.79225477406022	0.06499673017632
C	-2.57254180930988	0.68066924697672	0.04014779830975
C	-3.82541119774996	0.79960135418336	-0.57992837064325
H	-4.10726171852904	1.72503327485559	-1.07276605431871
N	-4.71126372750482	-0.18872186445604	-0.60481879475129
C	-4.35811891511048	-1.31523561770986	0.01174519659179
H	-5.06951781395205	-2.13393959035069	0.00230456385544
C	-3.13077966028134	-1.43557623652266	0.65724928847597
H	-2.86623937277249	-2.35064839389630	1.17793186557350
N	-2.24203262006466	-0.45056564177363	0.67820718837635
C	4.11161134183473	-0.06359651370539	0.96872751157952
H	4.10156167574320	0.68694407566941	1.75501969784740
H	4.81530012262442	0.21423285554487	0.18103745499750
H	4.40605514902859	-1.02378069418339	1.39046934643440
Br	3.80018867980812	-0.60362157351655	-2.58524396170535

iv). E-bpeMeBr

C	0.89179450361017	-0.16516932023592	-0.23714747767638
N	1.10798894069154	-0.39960072135889	-1.57890189184125
C	2.32493428074532	-0.41058859996503	-2.01141776217387
H	2.49752781967681	-0.57025163527571	-3.07007466540423
C	3.47112894034254	-0.19014946716875	-1.17346796775366
H	4.44944099376208	-0.50646737161911	-1.49366146912094
N	3.23380198344536	-0.21879188732387	0.16653613825108
C	1.95602948537898	-0.08600417198402	0.62288917621231
H	1.83418815839346	0.04151706981063	1.68847132254856
C	-0.46636786246401	-0.05051268394536	0.25459520045624
H	-0.58961712765392	0.11184905028857	1.31972112436150
C	-1.56296357459381	-0.12599207219715	-0.51551626831228
H	-1.44821291965245	-0.28019680259028	-1.58196234615115
C	-2.92458166862358	-0.01023218216642	-0.00966140933160
C	-4.01566138103874	-0.08948192458561	-0.89048624180600
H	-3.84915188812651	-0.23784994471070	-1.95293712298616
N	-5.27553816169932	0.01113799597362	-0.48506812871179
C	-5.46708186207011	0.19569152687923	0.81985332124307
H	-6.49038001558130	0.28042602336102	1.16873587508127
C	-4.39494868553083	0.27647606625890	1.70631385036081
H	-4.56498179075942	0.42662201264661	2.76794479349997

N	-3.13623393786246	0.17485263917542	1.30415733201227
C	4.36788548942406	-0.09022875426354	1.07760009513157
H	4.03599084785560	-0.27207178315370	2.09674050565017
H	4.78423024799870	0.91514266069140	0.98563467723887
H	5.13204106371079	-0.81995272921126	0.81226168935174
Br	4.09873712062106	2.08982600666992	-1.79115135013014

v). Z-bpeMeCl

C	0.64692552504400	0.74813996494994	-0.05258958052797
N	0.29717539415800	-0.38247143424774	-0.76720896629781
C	1.20122382551884	-1.26392487380336	-1.00500504190135
H	0.93272032126320	-2.14053885330033	-1.58559958618818
C	2.59400945407359	-1.13666961916919	-0.59445665302823
H	3.16184607098384	-2.04302881006872	-0.44222334347244
N	2.80720337681312	-0.16263211354583	0.35304645763612
C	1.89611640840063	0.84711438921395	0.48812746049037
H	2.20265421590201	1.68941170900593	1.09187972520712
C	-0.31063876376397	1.83684752925922	0.10927468077655
H	0.14861469011437	2.81171503856819	0.24287419682844
C	-1.65314686744529	1.81705674215627	0.07609201486929
H	-2.14786923858131	2.78327655154466	0.09531184095514
C	-2.57173142869445	0.67460743308336	0.04403515617356
C	-3.81505325775563	0.80032133218134	-0.59542524595698
H	-4.08365718056345	1.72825187563494	-1.09115194149526
N	-4.70622285140017	-0.18234150006750	-0.63620679574367
C	-4.36917812233932	-1.31234112584633	-0.01600365152479
H	-5.08475329112736	-2.12722477215831	-0.03856480735622
C	-3.15343075349034	-1.43964895694734	0.64858393409126
H	-2.90231483256747	-2.35689932789299	1.17238633487134
N	-2.25923000924761	-0.45969147140051	0.68597059464696
C	4.13733513544830	-0.04296381221205	0.93799838575065
H	4.09871652608716	0.63255349337501	1.78969191161494
H	4.84011929448636	0.33309174201129	0.19188888175515
H	4.47377259259704	-1.02257574048444	1.27742837314624
Cl	3.61879376608593	-0.62943538983943	-2.38015533532020

vi). E-bpeMeCl

C	0.90094103380812	-0.16365149861584	-0.23352964766619
N	1.13031139366605	-0.43190107221791	-1.57450272099360
C	2.33696607473680	-0.40353036281631	-2.00949356560924
H	2.51941700462310	-0.61260421684072	-3.05887615767183
C	3.49456025577849	-0.06054719014246	-1.18185238811768
H	4.45068511111135	-0.46455421317669	-1.48469280748889
N	3.24584451417743	-0.16668567784848	0.17233766588333
C	1.96008458448663	-0.05878133778124	0.62264275763512
H	1.83154285151309	0.06249831107094	1.68877980012976
C	-0.46105091210230	-0.06006977434793	0.24490124422869
H	-0.59155007587906	0.12892625247189	1.30506481662097
C	-1.55627744165953	-0.16358945258681	-0.52499989113048
H	-1.43969801722107	-0.34513332130563	-1.58688653189567
C	-2.91816822119998	-0.03509299253287	-0.02482743702073

C	-4.00944600001919	-0.14602328708259	-0.90373135720350
H	-3.84069549456375	-0.33045396882621	-1.96025422514200
N	-5.26976874978094	-0.03394339418287	-0.50459212232174
C	-5.46483774182824	0.19388437195746	0.79362236510552
H	-6.48902597303757	0.28892223194472	1.13723100057563
C	-4.39449203378566	0.30549492352056	1.67768036299390
H	-4.56653078668156	0.49051361276346	2.73363674373402
N	-3.13432116646443	0.19315003669303	1.28172893148517
C	4.36703436402597	-0.04220439983064	1.09554782167734
H	4.05343770665956	-0.35343392391328	2.08967022861745
H	4.72114930659708	0.99043267635561	1.12295030897465
H	5.18206466260310	-0.68558326099926	0.76410523456620
Cl	3.94182275043650	1.90396192827007	-1.68166142996621

vii). Z-bpeMeF

C	0.64052813154502	-0.15772455913055	1.47632382331603
N	1.72052426799858	0.14611279251123	2.29746165293378
C	2.91212722222424	0.00051818392470	1.85234504414113
H	3.74596743052836	0.28396140706379	2.48816722777047
C	3.24155802134858	-0.60658044054732	0.53187259066589
H	4.13162136111256	-0.18828212004868	0.05972198274928
N	2.13277880021188	-0.55098613502936	-0.33876543801857
C	0.87058944212326	-0.48912193960337	0.17555543669837
H	0.07067004915624	-0.68220681086578	-0.52354481067738
C	-0.66832865100419	-0.11275767770203	2.12648656654534
H	-0.58972358762241	-0.29983815229271	3.19300669328459
C	-1.91270687000149	0.08229155721821	1.65522689373114
H	-2.72740320082511	-0.07195850750306	2.35439353233254
C	-2.38533657263956	0.46751398087873	0.32015734493803
C	-1.70736609992915	1.37155865039670	-0.51604420757908
H	-0.77227489429575	1.81642543275391	-0.19555858477421
N	-2.17128001657388	1.73946504715311	-1.70248984481977
C	-3.34132989111808	1.21719867068502	-2.07776413657826
H	-3.72845114811945	1.50480903133336	-3.04904150448575
C	-4.04475922932935	0.35648848714321	-1.24545767907186
H	-5.01157967728403	-0.03837721875302	-1.54151156228599
N	-3.58121528983421	-0.01328589745609	-0.05522241176865
C	2.34526202719239	-0.88938844022455	-1.74099879131049
H	1.49316655348694	-0.55345742896053	-2.32913945711459
H	2.48000688263084	-1.96464105696729	-1.87691348433468
H	3.23925093647697	-0.37865610648755	-2.10124721315993
F	3.61770600254078	-1.98907774949006	0.76297933687262

viii). E-bpeMeF

C	0.93720541322235	-0.13230811858853	0.10371803714396
N	1.57541033559704	0.69363483044508	1.02002588829666
C	2.76441057113777	0.40669504435550	1.39360027167117
H	3.27622402246073	1.08120857830214	2.07452116241717
C	3.48937130602066	-0.83497556409062	0.99182095919408
H	4.56945484046050	-0.70477950087266	0.91180772035936
N	2.95667419238692	-1.36140740332993	-0.20376602304885

C	1.64985017039990	-1.11857938257504	-0.51445414346787
H	1.22629352212443	-1.71592257383745	-1.31041089168278
C	-0.44964902372880	0.11199684224666	-0.22523027306224
H	-0.89438063933980	-0.54433556114119	-0.96609671113477
C	-1.22505504243114	1.05785488272507	0.33063298199003
H	-0.79999182245856	1.71721710090931	1.07809836975947
C	-2.63250037476496	1.24721588040806	0.01203807055796
C	-3.38014633185589	2.24137572722911	0.66912798391910
H	-2.90613041200891	2.87332610350610	1.41400207501038
N	-4.66599360923382	2.45612129194520	0.42627805207273
C	-5.23778928869550	1.67397320255256	-0.48955576540618
H	-6.28917257072654	1.83522715150855	-0.70077991145375
C	-4.51228968080521	0.68679251039671	-1.15035926677173
H	-4.98860995207076	0.05609717080670	-1.89511600876972
N	-3.22644996437946	0.46940317417154	-0.90956289724571
C	3.70380772778395	-2.40425420936091	-0.89415378750143
H	3.33087653787637	-2.50435962755572	-1.91221108118987
H	3.61915638877858	-3.36575719074771	-0.38231906977118
H	4.75722104826378	-2.12453289496902	-0.93658366598729
F	3.35220263598640	-1.79692746443951	2.06492992410132

ix). Z-bpeMe

C	0.74671515742333	-0.23123056932664	1.55781943803772
N	1.81632664476335	0.08941367364792	2.30414879251812
C	3.01767493974152	0.08745385335735	1.74352309742830
H	3.86178562804174	0.36149048991316	2.36518979678244
C	3.22076443191350	-0.25302957925403	0.41682726000530
H	4.18780604725850	-0.26088521296556	-0.06241677191715
N	2.15020525854214	-0.61584485074352	-0.31842798694326
C	0.92600337072730	-0.61661748235076	0.22089985657540
H	0.11402167931701	-0.94478967288134	-0.40930308490340
C	-0.54908473716861	-0.26090640754018	2.23052399615866
H	-0.46156915457698	-0.47053781109604	3.29043008039007
C	-1.77721800589432	-0.06619210364561	1.72234548221352
H	-2.61543099860907	-0.24375668242680	2.38812951121450
C	-2.17793437948456	0.38090564406632	0.38050298958814
C	-1.52043157874256	1.40137851959913	-0.31596619231984
H	-0.68498463437074	1.93350282113804	0.12828691881344
N	-1.90832735883231	1.79614036910409	-1.52452315992487
C	-2.98103079746158	1.19867981969208	-2.03621021674791
H	-3.30419034499531	1.51237236419646	-3.02227470653086
C	-3.67763537536135	0.21867293812018	-1.32523146325219
H	-4.57269889625606	-0.23805591615709	-1.73276635594180
N	-3.28147028096256	-0.18809762998008	-0.12691676995033
C	2.33407494050189	-1.06687470692904	-1.71861711600352
H	1.47525180505673	-0.75546958992451	-2.30677903340274
H	2.42425043444178	-2.15238408768972	-1.73047783847430
H	3.23712720498722	-0.61533718992380	-2.11871652341345

x). E-bpeMe

C	1.05353101823196	-0.14356072365350	0.21441911085309

N	1.62959739088251	0.64572040283802	1.14213854168439
C	2.89709648598832	0.44359500411637	1.45655109251034
H	3.33836311552361	1.09125608156340	2.20520828156243
C	3.66927576880939	-0.55158103242851	0.87336755331999
H	4.70486519921787	-0.73386689084035	1.11707660470540
N	3.09262543147401	-1.34082813360254	-0.05521581310160
C	1.81321720121576	-1.16054416177223	-0.39538016308989
H	1.40152451232312	-1.82056031418757	-1.14462946477403
C	-0.32691616444407	0.03247130210509	-0.16205844190151
H	-0.73536755954095	-0.64102660107392	-0.90604836962211
C	-1.13059585129672	0.96845657882955	0.38095259188006
H	-0.72674837917322	1.64518767202483	1.12565140479209
C	-2.53547443697720	1.12407054163686	0.04563714666743
C	-3.30321532850536	2.11829195995459	0.66362251972811
H	-2.86101286881419	2.78450181054417	1.39768843367236
N	-4.59223601563253	2.29250380842812	0.38693195913194
C	-5.12155318285161	1.47178257026138	-0.51199534023127
H	-6.17174536737158	1.60148966567088	-0.74873985789655
C	-4.36249951901175	0.47248992353715	-1.13739919899488
H	-4.81316089691906	-0.18883514289943	-1.86941958310464
N	-3.08230148227197	0.29767020839634	-0.86426273650508
C	3.89597963964071	-2.41090011101466	-0.69056421671733
H	4.74741906824579	-1.95943711124585	-1.19613443482858
H	3.27838648320056	-2.94162237535635	-1.40816855164453
H	4.24094573805656	-3.09672693183185	0.08077293190437
